# EGF-conditioned M1 macrophages Convey reduced inflammation into corneal endothelial cells through exosomes

**DOI:** 10.1016/j.heliyon.2024.e26800

**Published:** 2024-02-22

**Authors:** Soo Jin Lee, Seung Hyeun Lee, Ahra Koh, Kyoung Woo Kim

**Affiliations:** a Chung-Ang Ocular Surface Restoration via Immune-inflammation Alleviation (CORIA) Laboratory, Seoul, Republic of Korea; b Department of Ophthalmology, Chung-Ang University College of Medicine, Chung-Ang University Hospital, Seoul, Republic of Korea; c Chung-Ang University Graduate School, Republic of Korea

## Abstract

Epidermal Growth Factor (EGF), a protein pivotal in cell proliferation and survival, has recently shown promise in alleviating inflammation. This study investigates EGF's impact on M1 macrophages, exploring its potential for anti-inflammatory and anti-vasculogenic interactions with corneal endothelial cells (CECs). Polarized M1 macrophages treated with EGF exhibited a suppression of gene expressions related to inflammatory and vasculogenic signals. The anti-inflammatory effects of EGF were observed in co-culture systems with human CECs (HCECs), showcasing its ability to alter macrophage phenotypes. Exosomes derived from EGF-treated M1 macrophages demonstrated enriched proteomic profiles related to immune system regulation and inflammation inhibition. When applied as eye drops in murine corneas, EGF-conditioned M1 macrophage-derived exosomes effectively reduced inflammation and increased M2-related *ARG1* expression. This study highlights EGF's potential in mitigating inflammation in M1 macrophages and its delivery through exosomes to cultured HCECs and murine corneas, suggesting a novel therapeutic avenue for ocular surface anti-inflammatory treatments.

## 1 Introduction

The eye has long been recognized as one of the well-known immune-privileged organs [1]. To maintain ocular immune privilege, particularly in the cornea, the absence of blood vessels is a normal feature [2]. Additionally, the corneal endothelium serves as a barrier between the corneal stroma and the chamber, expressing surface molecules that inhibit complement activation, lymphocyte activation, and inflammation [[3], [4], [5]]. However, the conjunctiva is equipped with mucosa-associated lymphoid tissue, known as conjunctiva-associated lymphoid tissue, making it easily susceptible to immunological activation [6]. Immune cells also exist on the human cornea and they play a crucial role in mucosal immune responses to prevent infections and damage [7]. Recent advancements, such as single-cell RNA sequencing, have identified various immune cell types in the corneal limbus, including CD8^+^ T cells, naïve T cells, double-negative T cells, as well as innate cells like macrophages, dendritic cells, monocytes, and basophils [8].

Among innate immune cells, macrophages form a diverse organ system across the body, acting as phagocytes to defend against pathogens and remove dead cells [9]. CD11b^+^ macrophages have been highlighted as the primary resident immune cells in the cornea, distributed from the central cornea to the peripheral limbus [10], constituting roughly 50% of all immune cells in the cornea [11]. In healthy murine corneas, a CD11c^−^CD11b^+^ population of bone marrow-derived cells, primarily representing monocytes/macrophages, is found in the posterior stroma [12]. Corneal resident myeloid-lineage cells primarily recognize and mediate host responses to toll-like receptor (TLR) ligands through their pattern recognition receptors [13]. For instance, exposure to the TLR9 ligand leads to macrophage accumulation on the corneal endothelium, forming multinucleated giant macrophages [14]. This phenomenon, known as keratic precipitate, is a common sign of corneal stromal or endothelial inflammation in conditions like herpes simplex keratitis and uveitis [15].

Epidermal growth factor (EGF) is a well-known protein that stimulates cell proliferation, differentiation, and survival [16]. Its role in tissue recovery after damage through EGF receptor (EGFR) signaling has been extensively studied, especially in skin [17]. In the cornea, the distribution of EGFR in corneal epithelial cells has been reported [18]. The presence of high basal levels of EGF in tear fluid suggests its importance in corneal epithelium restoration and homeostasis [19]. However, the hypothetical role of EGF in the immune system remains controversial. EGFR activation has been linked to accelerated colitis and impaired disease recovery due to inhibition of interleukin (IL)-10 production [20]. Conversely, heparin-binding EGF-like growth factor (HB-EGF) has been shown to decrease M1 polarization induced by lipopolysaccharide (LPS) and promote M2 polarization while protecting human fetal small intestinal epithelial FHs-74 cells [21].

Although corneal endothelial cells (CECs) are known to express EGFR *ex vivo*, the role of EGF in the interaction between macrophages and CECs has yet to be elucidated. This study aims to investigate whether EGF modulates M1 macrophages to facilitate anti-inflammatory and anti-vasculogenic interactions with CECs.

## 2 Results

### 2.1 Human THP-1 monocyte polarization into M1 macrophages

**Fig. 1:**
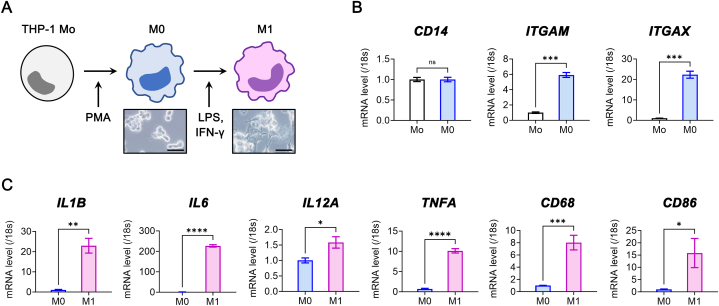
M1 macrophage polarization from THP-1 human monocyte cell line. (**A**) Scheme of stepwise polarization process and the representative M0 and M1 macrophages. Scale bars: 50 μm. Mo, monocyte; PMA, phorbol 12-myristate 13-acetate; LPS, lipopolysaccharide; IFN-γ, interferon gamma. (**B–C**) Real-time RT-PCR analysis for *CD14*, *ITGAM* (i.e. *CD11B*), *ITGAX* (i.e. *CD11C*), *IL1B*, *IL6*, *IL12A*, *TNFA*, *CD68*, and *CD86* genes in M0 and M1 macrophages (*N* = 3 to 5). *P< 0.05, **P< 0.01, ***P< 0.001, ****P< 0.0001, ns: not significant.

We differentiated human THP-1 monocytes into M0 macrophages using PMA, followed by polarization into M1-like macrophages with LPS and IFN-γ (Fig. 1A). During M0 differentiation, *ITGAM* (i.e. *CD11B)* and *ITGAX* (i.e. *CD11C)* gene expression increased (Fig. 1B). Subsequently, genes associated with inflammatory cytokines (*IL1B, IL6, IL12A, TNFA*) and M1 surface markers (*CD68, CD86*) were upregulated during M0-to-M1 polarization (Fig. 1C).

We gated the CD14^mod to hi^ cell population and the CD14^lo^ cell population isolating a CD86^+^CD206^-^ subset representing potential M1 macrophages [22]. Unlike THP-1 monocytes, CD14^lo^CD86^+^CD206^-^ and CD14^+^CD86^+^CD206^-^ cells increased in the polarized M1 macrophage population through flow cytometric analysis (Fig. S1).

### 2.2 EGF suppresses inflammatory and vasculogenic signals in M1 macrophages

**Fig. 2:**
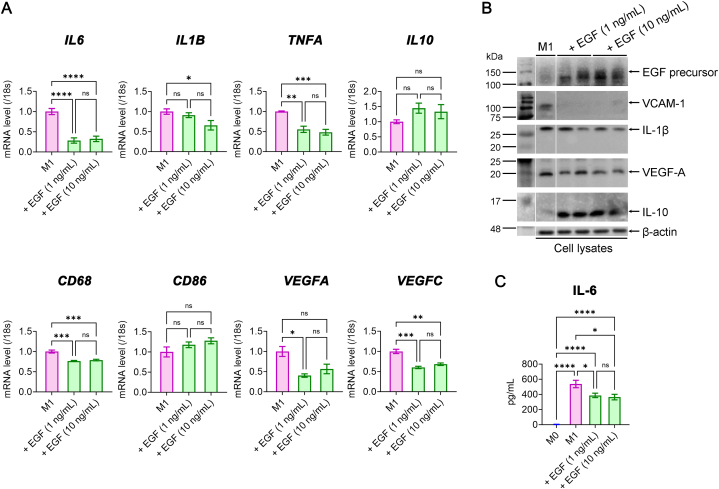
EGF-induced alteration of inflammatory and vasculogenic signals in M1 macrophages. (**A**) Real-time RT-PCR analysis for *IL6, IL1B, TNFA, IL10, CD68, CD86, VEGFA,* and *VEGFC* genes with and without EGF treatment in M1 macrophages (*N* = 4 to 7). (**B**) The representative Western blot image for EGF precursor, VCAM-1, IL-1β, VEGF-A, and IL-10 protein in M1 macrophages with and without EGF treatment. The small gaps indicate skipped lanes from the same membrane. Full-length gels before cropping are noted in Fig. S4. (**C**) The result of ELISA for IL-6 levels in M0, M1, and M1 with EGF treatment (*N* = 7). *P< 0.05, **P< 0.01, ***P< 0.001, ****P< 0.0001, ns: not significant. EGF, epidermal growth factor; IL, interleukin; VEGF, vascular endothelial growth factor; VCAM-1, vascular cell adhesion molecule 1.

To assess the influence of exogenous EGF on M1 macrophage inflammatory and vasculogenic activity, we treated M1 macrophages with human recombinant EGF at concentrations of 1 ng/mL and 10 ng/mL for 12 h. We observed reduced gene expression of inflammatory markers (*IL6, IL1B, TNFA*), phenotypical marker of macrophage (*CD68*) and vasculogenesis-related factors (*VEGFA* and *VEGFC*) (Fig. 2A). At the protein level, EGF treatment downregulated VEGF-A, VCAM-1, and IL-1β while upregulating the anti-inflammatory cytokine IL-10. This coincided with increased intracellular EGF precursor protein levels in M1 cell lysates (Fig. 2B). ELISA analysis further confirmed reduced IL-6 secretion following EGF treatment (Fig. 2C).

### 2.3 EGF reduces inflammatory signals in corneal endothelial cells during co-culture with M1 macrophages

**Fig. 3:**
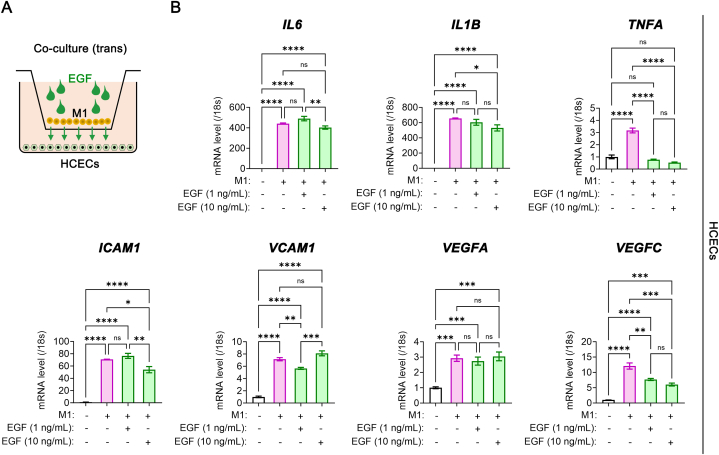
EGF-induced attenuation of inflammatory signals in HCECs during co-culture with M1 macrophages. (**A**) Scheme of co-culture of M1 macrophages and HCECs using transwell system with EGF treatment. (**B**) Real-time RT-PCR analysis for *IL6, IL1B, TNFA, ICAM1, VCAM1, VEGFA* and *VEGFC* genes in HCECs with and without EGF during co-culture with M1 macrophages (*N* = 4). *P< 0.05, **P< 0.01, ***P< 0.001, ****P< 0.0001, ns: not significant. EGF, epidermal growth factor; human corneal endothelial cells, HCECs.

CECs are vital for maintaining corneal clarity and proper function [23]. In the corneal stroma, macrophages are nearby and contribute to various functions, including immune responses and wound healing. This geometric arrangement enables them to contribute to innate immune responses, facilitate efficient wound healing, and uphold vessel homeostasis under steady-state conditions [12,24]. On the contrary, macrophages have been implicated in mediating corneal endothelial rejection following corneal transplantation [25]. We investigated EGF's anti-inflammatory effects in a co-culture system involving M1 macrophages and human CECs (HCECs) (Fig. 3A).

Co-culturing HCECs with M1 cells led to increased gene expression of pro-inflammatory cytokines (*IL6, IL1B, TNFA*) and adhesion molecules (*ICAM1 and VCAM1*), along with elevated expression of vasculogenic growth factors (*VEGFA* and *VEGFC*) in HCECs (Fig. 3B). However, when treated with EGF for 24 h, EGF notably suppressed the enhanced gene expression in HCECs, except for *IL6* and *VEGFA* (Fig. 3B).

### 2.4 EGF modifies proteome composition in M1 macrophage-derived exosomes

Exosomes are small (40–160 nm) vesicles that facilitate cell-to-cell communication by delivering various signals [26]. In a prior study, EGFRs were observed to be internalized in *ex vivo* CECs following EGF stimulation, suggesting a potential feedback inhibition mechanism for EGF functions in ocular surface cells [27]. Hence, we conducted a proteome analysis on M1 macrophages to investigate if exosomes derived from EGF-conditioned M1 macrophages (EGF-M1 *exo*) carried inflammation-relieving signals compared to those from untreated M1 macrophages (M1 *exo*). Our aim was to screen proteins that might contribute to EGF-M1 *exo*'s efficacy in alleviating inflammation.

**Fig. 4:**
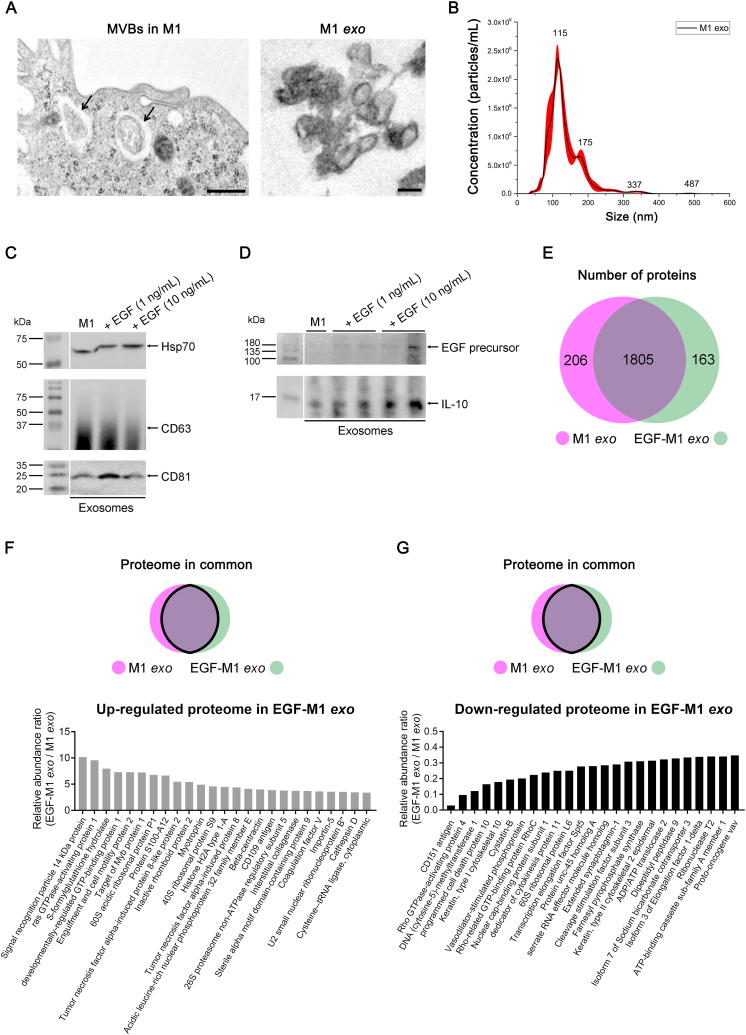
Verification of M1 macrophage-derived exosomes and proteomic analysis in exosomes. (**A**) Representative transmission electron microscope photographs of multivesicular bodies (MVBs, arrows) and released exosomes from M1 macrophage (M1 *exo*). Scale bar: 500 nm. (**B**) Size and concentration of M1-derived exosomes evaluated by particle tracking analysis method. (**C-D**) The representative Western blot image for exosomal markers including Hsp70, CD63, CD81, and EGF precursor and IL-10 protein in M1 macrophage-derived exosomes with and without EGF treatment. The small gaps indicate skipped lanes from the same membrane. Full-length gels before cropping are noted in Fig. S5. Cropped gels are accompanied by full-length gels. (**E**) The number of proteins in each M1 *exo* and EGF-conditioned M1 macrophage-derived exosome (EGF-M1 *exo*) according to proteomic analysis of exosomes by mass spectrometry. (**F-G**) Most upregulated (**F**) or downregulated (**G**) 25 proteins among exosomal proteome in common from M1 *exo*s and EGF-M1 *exo*s based on the relative abundance of exosomal proteins. EGF, epidermal growth factor; IL-10, interleukin 10.

We began by examining exosomes in both M0 and M1 macrophages (Fig. 4A) and then isolating and verifying exosomes from M1 macrophages based on their size and the presence of exosomal markers like Hsp70, CD63, and CD81 (Fig. 4B and C). EGF treatment increased the exosomal expression of EGF precursor and the anti-inflammatory cytokine IL-10 in M1 macrophages (Fig. 4D). Next, we conducted a proteome analysis on M1-derived exosomes to identify compositional changes induced by EGF treatment. In total, we identified 2174 proteins in each exosome preparation. Of these, 2011 proteins were in M1 *exo*, and 1968 proteins were in EGF-M1 *exo*, with 1805 proteins common to both (Fig. 4E).

To understand the biological significance of these specific proteomes, we identified the top 25 proteins with the highest up-regulation and down-regulation in relative abundance from the common proteome pool. Proteins more abundant in EGF-M1 *exo*s included ras GTPase-activating protein 1, TNF-α-induced protein 8-like protein 2, and inactive rhomboid protein 2 (IRP2), which are known to negatively regulate apoptotic processes, T cell activation, and the inflammatory response to antigenic stimuli, respectively, within the Gene Ontology (GO) biological process category (Fig. 4F). In contrast, proteins down-regulated by EGF in exosomes included CD151 antigen, elongation factor 1-delta, and ribonuclease T2, which are known to play roles in T cell proliferation, positive regulation of I-kappaB kinase/NF-kappaB signaling, and the innate immune response, respectively (Fig. 4G).

We also conducted pathway analysis for proteins unique to M1 *exo* (206 proteins) and EGF-M1 *exo* (163 proteins) using Reactome pathway analysis. Interestingly, the heme degradation pathway, known for its role in regulating TNF-α and suppressing LPS-induced IL-6 production [24], was most highly involved in EGF-M1 *exo*'s unique proteome pathways (Fig. S2).

Furthermore, we conducted a screening among whole exosomal proteome focused on proteins associated with immunological functions, utilizing categories like biological processes in GO, Wiki pathways, and Reactome pathways. In M1 *exo*-specific proteome, proteins including interferon regulatory factor 3, tyrosine-protein phosphatase non-receptor type 11, interferon regulatory factor 2-binding protein 2, TNF, and VCAM-1, which are involved in immune system activation, were found (Fig. S3A). In EGF-M1 *exo*-specific proteome, collagen alpha-2(I) chain, superoxide dismutase, transforming growth factor beta-1, and signal transducer and activator of transcription 2, which have role in negative regulation of inflammatory- and immunologic signals, were found (Fig. S3B).

Among the targets where changes in mRNA levels were confirmed in both M1 macrophages and HCECs in Fig. 2, Fig. 3, the relative abundance of IL-1β and ICAM-1 in the exosomal proteome pool decreased with EGF treatment, showing ratios of 0.30 (EGF-M1 *exo*/M1 *exo*) and 0.71, respectively. VCAM-1 was notably absent in EGF-M1 *exo* but exhibited high presence in M1 *exo*. VEGF-A, PDGFA, and MMP-2 were highly prevalent in both M1 *exo* and EGF-M1 *exo*, making the difference in their existence levels equivocal.

### 2.5 EGF-conditioned M1 macrophage-derived exosomes alleviate inflammatory signals in HCECs

**Fig. 5:**
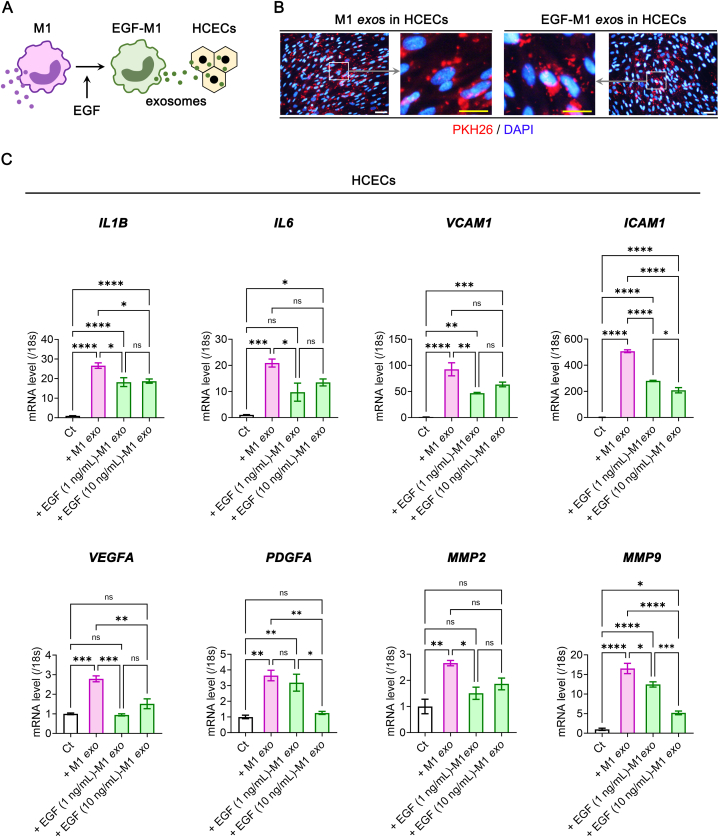
The induction of inflammatory and vasculogenic signals in HCECs by the delivery of M1 macrophage-derived exosomes and the relative attenuation of those signals by EGF-conditioned M1 macrophage-derived exosomes. (**A**) Scheme of delivery of M1 macrophage-derived or EGF-conditioned M1 macrophage-derived exosomes into the cultured HCECs. (**B**) The low-magnified and high-magnified immunofluorescence images of the delivered PKH26 dye-labeled M1 macrophage-derived exosomes (M1 *exo*s) and EGF-conditioned M1 macrophage-derived exosomes (EGF-M1 *exo*s) delivered and penetrated into the cultured HCECs. Scale bars: 100 μm (white) and 50 μm (yellow). (**C**) Real-time RT-PCR analysis for *IL1B, IL6, VCAM1, ICAM1, VEGFA, PDGFA, MMP2* and *MMP9* genes in HCECs before and after M1 *exo* or EGF-M1 *exo* treatment (*N* = 4). *P< 0.05, **P< 0.01, ***P< 0.001, ****P< 0.0001, ns: not significant. EGF, epidermal growth factor; Ct, control; HCECs, human corneal endothelial cells.

Considering the inflammation-reducing effects of EGF in M1 macrophages and the changes in M1-derived exosome composition (Fig. 2, Fig. 4), we compared the impact of M1 *exo* and EGF-M1 *exo* on inflammatory and vasculogenesis-related signals in HCECs. Remarkably, exosomes derived from M1 macrophages effectively penetrated cultured HCECs (Fig. 5A and B). M1 *exo* significantly increased the expression of genes associated with inflammatory cytokines (*IL1B* and *IL6*), adhesion molecules (*VCAM1* and *ICAM1*), and vasculogenesis-related factors (*VEGFA, PDGFA, MMP2*, and *MMP9*) in HCECs (Fig. 5C). However, the administration of EGF-M1 *exo* at two different conditioning concentrations (1 ng/mL and 10 ng/mL) effectively counteracted the upregulation of all these targets induced by M1 *exo* in HCECs (Fig. 5C).

### 2.6 EGF-conditioned M1 macrophage exosome eye drops alleviate inflammation and vasculogenic signals in murine cornea

**Fig. 6:**
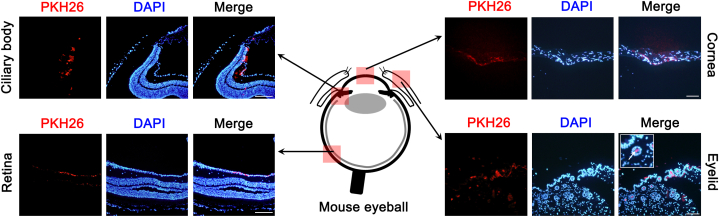
The intraocular delivery of the exosome eye drops in corneal epithelium removed murine mouse model. The existence of PKH26 dye-labeled exosome verified by the immunofluorescence stain in various ocular areas, including the cornea, ciliary body, retina, and eyelid after 3 regular applications of M1 macrophage-derived eye drops (30 μg/5 μL per 1 drop) over 24 h. In the eyelid, the delivered exosomes from eye drops were delivered into the glandular system of the eyelid (arrow). Scar bars: 200 μm.

We further evaluated the effects of M1-derived exosomes on murine eyes. First, we investigated the absorption of exosome eye drops on the ocular surface and within the eye. After applying M1 *exo* eye drops three times over 24 h, we detected fluorescence from the applied M1 *exo*s in various ocular areas, including the cornea, ciliary body, retina, and eyelid (Fig. 6). Notably, exosomes were successfully delivered into the eyelid's glandular system.

**Fig. 7:**
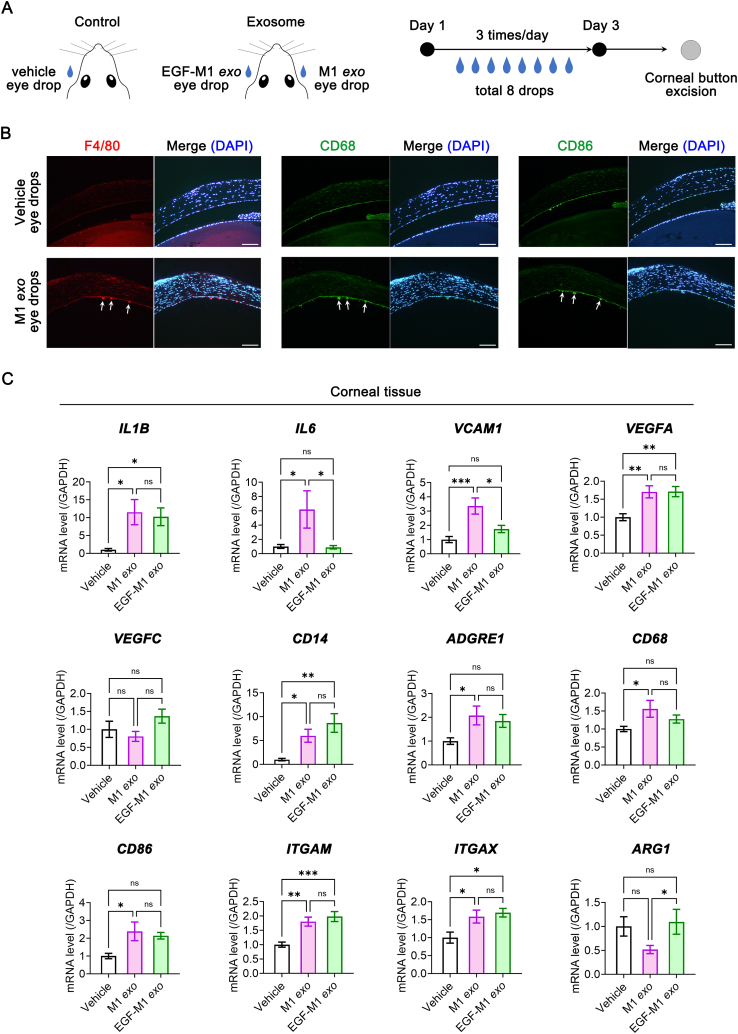
The alteration of inflammatory and vasculogenic signals corneal tissue after the application of eye drops made of EGF-conditioned M1 macrophages in corneal epithelium removed murine mouse model. (**A**) The scheme of animal experiments. The Balb/c mice were divided into control and exosome groups. In control group, the vehicle eye drops were applied in the unilateral eye after the corneal epithelial peeling. In exosome group, the M1 macrophage-derived exosome (M1 *exo*) eye drops and EGF-conditioned M1 macrophage-derived exosome (EGF-M1 *exo*) eye drops were applied in right and left eye, respectively, after the corneal epithelial peeling. The eye drops were applied 3 times a day and total 8 drops. (**B**) The immunofluorescene stain of pan-macrophage markers including F4/80 and CD68, and CD86 as a M1 macrophage marker in the excised corneal tissues after vehicle or M1 *exo* eye drops. The F4/80-, CD68^−^or CD86-positive cells are observed in the surface of cornela endothelium (arrows) after the application of M1 *exo* eye drops. Scale bars: 200 μm. (**C**) Real-time RT-PCR analysis for genes related with inflammatory and vasculogenic signals including *IL1B, IL6, VCAM1, VEGFA,* and *VEGFC*, and genes related with monocyte/macrophage markers including *CD14, ADGRE1, CD68, CD86, ITGAM* (i.e. *CD11B*), *ITGAX* (i.e. *CD11C*), and *ARG1* (*N* = 10). *P< 0.05, **P< 0.01, ***P< 0.001, ns: not significant. EGF, epidermal growth factor.

Next, we compared the effects of M1 *exo* and EGF-M1 *exo* on inflammatory signals in murine eyes. Using C57BL/6 mice, we removed the corneal epithelium and administered M1 *exo* and EGF-M1 *exo* eye drops a total of eight times. Subsequently, we excised the corneal buttons for analysis (Fig. 7A). After treatment with M1 *exo* eye drops, we observed cells expressing F4/80, CD68, or CD86 on the corneal endothelium, indicating keratic precipitate formation (Fig. 7B). M1 *exo* eye drops increased the gene expression of inflammatory and vasculogenic factors (*IL1B, IL6, VCAM1*, and *VEGFA*) in the excised corneal tissues. In contrast, EGF-M1 *exo* eye drops did not induce the gene expression of *IL6* and *VCAM1* (Fig. 7C).

Both M1 *exo* and EGF-M1 *exo* eye drops elevated the gene expression of macrophage markers *CD14, CD11B,* and *CD11C* in corneal tissues. However, gene expressions of *CD68*, *CD86*, and *ADGRE1* which is associated with F4/80, were not upregulated by EGF-M1 *exo* eye drops. Intriguingly, the *ARG1* gene expression, linked to arginase-1 and indicative of anti-inflammatory M2 macrophages, significantly increased in response to EGF-M1 *exo* eye drops compared to M1 *exo* eye drops (Fig. 7C).

## 3 Discussion

Our results demonstrate the anti-inflammatory and anti-vasculogenic effects of EGF on M1 macrophages and its impact on HCECs and the murine cornea. Specifically, conditioning M1-polarized macrophages with EGF suppresses their intracellular inflammatory and vasculogenic signals and alters the immunological composition of exosomes in M1 macrophages. Consequently, while the direct application of EGF to HCECs during co-culture with M1 macrophages yielded unsatisfactory results, delivering EGF-conditioned M1-derived exosomes into HCECs significantly attenuated inflammatory and vasculogenic signals. Fortunately, the effect of EGF-M1 *exo* eye drops partially differed from the effect of M1 *exo* eye drops in the murine cornea. This difference was represented by the attenuation of *IL6* and *VCAM1* gene expression and activation of *ARG1* gene expression.

In induced M1 macrophages from THP-1 monocytes, stimulating EGFR of M1 macrophages using EGF, a prototype ligand of EGFR, downregulated inflammatory and vasculogenic signals. While EGFR is present on human macrophages [28], its role in macrophages has mainly been studied in cancer research. However, EGFR has been found on rabbit peripheral monocytes and macrophages, where it promoted cell proliferation and migration [29]. Although we did not investigate the signal directly stimulating EGFR in M1 cells, it is likely that EGF exerts anti-inflammatory mechanisms in M1 cells derived from THP-1 cells.

Unfortunately, the direct application of EGF in HCECs did not significantly downregulate *IL6* gene expression. In contrast, EGF-M1 *exo* treatment in HCECs effectively suppressed *IL6* gene expression compared to M1 *exo* treatment. This discrepancy in the effect of EGF suggests that EGFR-mediated signaling may be easily compensated for by a negative feedback mechanism. Indeed, EGFR internalization in *ex vivo* rat CECs occurred rapidly within 15–30 min after EGF stimulation [27].

While the application of EGF-M1 exosomes led to the suppression of *IL1B, IL6, VCAM1, ICAM1, VEGFA, PDGFA, MMP2,* and *MMP9* expressions, the pre-conditioning concentrations of EGF required for inhibiting inflammatory or vasculogenic signals varied across targets. The reason for this variation remains unknown, but we hypothesize that compensatory signals triggered by EGF may differ among the targeted molecules. Macrophages are known to closely associate with adjacent tissue cells in response to reciprocal growth factor signals, including EGF, in various tissues, even in a steady state [30]. Considering the importance of growth factors in maintaining homeostatic interactions between macrophages and tissue cells, the optimal EGF concentration may be contingent upon the microenvironmental conditions of inflammation. Further investigation into this issue is warranted for future studies.Following EGF conditioning of M1 macrophages, we observed an increased amount of IRP2 among exosomal proteomes. We consider IRP2 as a possible representative of the EGF-related anti-inflammatory mechanism in M1 macrophages, as IRP2 is involved in the "CD163 mediating an anti-inflammatory response" pathway and interacts with and regulates a disintegrin and metalloprotease-17 (ADAM17) protease [31]. ADAM17 plays a crucial role in the proteolytic release and activation of several ligands of EGFR, as well as the IL-6 receptor [32]. Analyzing changes in IRP2 and ADAM17 expression during EGFR stimulation or in EGF-conditioned macrophages could be an interesting topic for future research.

Topical instillation of eye drops made from chemicals often has limitations, such as the need for frequent application and low bioavailability. Exosome eye drops may offer a solution to these problems due to their high penetration capability through barriers. In this study, only three regular exogenous applications of exosome eye drops penetrated the epithelium-peeled cornea and were easily delivered to the ciliary body and retina. Similarly, salivary gland epithelial cell-derived exosome cargos were loaded with autoantigens Ro/SS-A, La/SS-B, and Smith ribonucleoprotein, which were then used to immunologically induce Sjogren's syndrome [33].

While M1 *exo* eye drops successfully induced inflammatory signals in mouse corneal tissue, unfortunately, EGF-M1 *exo* eye drops did not significantly ameliorate the upregulated expressions of inflammatory *IL1B*, macrophage markers including *ADGRE1* and *CD68*, M1 marker *CD86*, and myeloid cell markers *ITGAM* and *ITGAX*, except *IL6*. Since EGF treatment in M1 macrophages did not induce *trans*-polarization into anti-inflammatory M2 macrophages (data not shown), it suggests that EGF preconditioning did not *trans*-differentiate M1 macrophages into other subtypes of macrophges. We hypothesized that EGF-M1 *exo* eye drops might also induce inflammatory signals in murine corneas given that EGF-M1 *exo* was also originally derived from M1 macrophages in fact. Zhao et al. reported that the activation of EGFR in macrophages using HB-EGF mediates negative feedback inhibition of M2 polarization in Raw 264.7 cells and peritoneal macrophages [34]. On the contrary, HB-EGF significantly decreased LPS-induced M1 polarization and promoted M2 polarization via signal transducers and activators of transcription 3 activation in macrophages derived from THP-1 human monocyte cell lines [21]. Thus, the effect of EGFR stimulation may vary depending on the type of macrophages and the ligands of EGFR. While we were unable to discern the EGF-induced *trans*-differentiation of M1 macrophages into anti-inflammatory macrophages, the lack of upregulation of *ADGRE1, CD68*, and *CD86* as well as the activation of *ARG1* in murine corneal tissues after EGF-M1 *exo* eye drop application, suggests that EGF-conditioned macrophages might deliver signals capable of modulating the phenotype of M1 macrophages *in vivo*. This modulation appears to deactivate the M1 phenotype, particularly *in vivo*.

In murine corneal tissues, the application of EGF-M1 *exo* eye drops did not induce IL6 activation, in contrast to *IL1B*, which was activated even with EGF-M1 *exo* eye drops. Furthermore, *IL6* suppression was observed in HCECs treated with EGF-M1 *exo*. However, in a co-culture system of HCECs and M1 macrophages, EGF treatment did not suppress *IL6* gene expression in HCECs. Despite the traditional acceptance of IL-6 as a pro-inflammatory cytokine secreted by macrophages, it is known to have both pro- and anti-inflammatory properties, depending on its classic versus *trans*-signaling [35]. Therefore, caution is warranted when interpreting the suppression of the *IL6* gene and IL-6 secretion by EGF in M1 cells, as it may not unequivocally induce anti-inflammation in corneal tissues or HCECs.

In conclusion, our findings demonstrate EGF's roles in ameliorating inflammation in M1 macrophages and its delivery through exosomes to cultured HCECs and murine corneas. This highlights that EGF's novel potential for anti-inflammatory therapies in the ocular surface.

### 3.1 Limitations of the study

We used the THP-1 human monocyte cell line to verify phenotypical alterations with EGF, which may not fully reflect the conditions of macrophages in the cornea. Additionally, we did not investigate the EGFR-dependent action of EGF on macrophages and HCECs, which limits our ability to provide a mechanistic explanation for the anti-inflammatory role of EGF on macrophages and its subsequent effects on HCECs. The modifications in the expressions of different inflammatory and vasculogenic factors were predominantly confirmed at the mRNA levels rather than the protein levels. Additionally, the resulting alteration in inflammation-triggered vasculogenesis formations with EGF was not validated. The optimal concentration for preconditioning M1 macrophages with EGF, leading to a significant suppression of inflammatory and vasculogenic targets through exosomes, has not been established. Nonetheless, there have been no previous studies investigating the novel role of EGF beyond epithelial wound healing on the ocular surface. Future experiments to confirm the possible anti-inflammatory target of EGF in corneal macrophages are necessary to understand EGF's specific role in the corneal innate immune system.

## 4 Methods

### 4.1 Study approval

We obtained approval from the Institutional Review Boards at Chung-Ang University Hospital (Approval No. 2106-006-465) for using cadaveric ocular tissues in corneal endothelial culture, following the principles of the Declaration of Helsinki. The Institutional Animal Care and Use Committee of Chung-Ang University approved the experimental protocol (IACUC approval No. 2020-00127), aligning with the ARVO Statement for the Use of Animals in Ophthalmic and Vision Research.

### 4.2 Macrophage polarization from THP-1 monocytes

We induced M1 macrophages from the THP-1 cell line (TIB-202, ATCC, Manassas, VA, USA) following an established protocol [36]. THP-1 cells were cultured in RPMI 1640 (WelGENE, Daegu, South Korea) with supplements. After seeding in 6-well plates and differentiating them into M0 macrophages with 50 nM phorbol 12-myristate 13-acetate (PMA; #P8139, Sigma-Aldrich, St Louis, MO, USA) for 24 h, we polarized them into M1 macrophages by exposing them to LPS (O111:B4, #L2630, 100 ng/ml, Sigma-Aldrich) and recombinant human interferon-gamma (IFN-γ, #570216, 20 ng/ml, BioLegend, CA, USA) for 24 h.

### 4.3 Culture of human corneal endothelial cells

We followed a previously established protocol for culturing human corneal endothelial cells (HCECs) [37]. Human corneal tissues without ocular diseases were obtained for transplantation and stored at 4 °C in Optisol-GS storage medium (Bausch & Lomb, Rochester, NY, USA). After excising the central 8-mm round cornea for transplantation, we utilized the peripheral corneal tissues for HCEC culture. These corneal tissues were thoroughly washed with phosphate-buffered saline (PBS, WelGENE) containing antibiotics (penicillin/streptomycin). To isolate HCECs, we carefully removed Descemet's membrane and corneal endothelium, followed by digestion with 1 mg/ml collagenase A (#07434, STEMCELL Technologies Inc, Vancouver, Canada) at 37 °C for 2 h. The detached HCECs were placed in corneal endothelial cell growth medium (Opti-MEM™-I, #31985070, Gibco, NY, USA) supplemented with various components. HCECs were collected after centrifugation at 1200 rpm for 5 min and plated on FNC-coated tissue culture dishes. Cells were cultured at 37 °C in a humidified 5% CO_2_ incubator and sub-cultured when reaching 80–90% confluency using TrypLE Express (Gibco).

### 4.4 Co-culture of M1 macrophages and HCECs

HCECs were seeded in 6 well-plates at a density of 1.5 × 10^5^ cells/well and cultured for 24 h. Co-culture were conducted using transwell inserts with a pore size of 0.4 μm (SPL Life Sciences, Pocheon-si, South Korea) placed into 6-well plates. M1 macrophages were resuspended in RPMI 1640 at density of 1 × 10^5^ cells/insert and then treated with EGF. The co-cultures of M1 macrophages and HCECs were incubated undisturbed at 37 °C in a humidified 5 % CO_2_ incubator for 24 h. RNA was subsequently extracted from HCECs (6-well plate) for analysis.

### 4.5 Extraction and verification of exosomes

M0 and M1 macrophages were cultured in RPMI 1640 medium containing exosome-depleted FBS (#EXO-FBS-250A-1, System Biosciences, CA, USA). Exosomes were precipitated from the cultured medium using the Total Exosome Isolation Reagent (#4478359, Invitrogen, CA, USA). Briefly, the medium was centrifuged at 2500 rpm for 30 min to remove cells and debris. The supernatant was collected and passed through a 0.22-μm-pore filter (#SLGVR33RS, Merck Millipore, MA, USA). Subsequently, the medium was mixed with the Total Exosome Isolation Reagent and incubated overnight at 4 °C. Exosomes were finally pelleted by centrifugation at 13,000 rpm for 1 h at 4 °C, re-suspended in PBS, and stored at −80 °C. The exosome protein content was quantified using a Pierce™ BCA protein assay kit (#23227, Thermo Fisher Scientific, Waltham, USA). The expression levels of exosomal marker proteins CD63, CD81, and heat shock protein 70 (Hsp70) were assessed using western blotting, as described in the Western blot analysis section.

The size distribution and concentration of purified exosomes were measured using nanoparticle tracking analysis with a Malvern NanoSight NS300 (Malvern Instruments Company, Malvern, England) and the corresponding software NanoSight NTA 3.4 Analytical.

### 4.6 Exosome staining and tracking

To label the exosomes, we used the PKH26 red fluorescent cell linker dye (#MINI26, Sigma-Aldrich) following the manufacturer's instructions. The exosomes were stained by adding 1 μL of PKH26 dye to 200 μL of Diluent C fluid from the kit and incubating them for 5 min at room temperature (RT). To halt the labeling process, we added an equal volume of 10 % BSA and re-purified the exosomes using the total exosome isolation reagent precipitation method. The culture medium of HCECs was replaced with medium containing PKH26-labeled M1-derived exosomes. The cells were incubated with these exosomes for 16 h and then fixed with 4 % paraformaldehyde after washing with PBS. Subsequently, we labeled the cells with Wheat Germ Agglutinin (WGA; #W11261, Invitrogen) for 30 min at RT and stained the nuclei with 4′,6-diamidino-2-phenylindole (DAPI). The cells were analyzed using an inverted fluorescence microscope (DMi8, Leica, Wetzlar, Germany).

### 4.7 EGF and exosome treatment

We treated M1 macrophages with recombinant human EGF (#E9644, Sigma-Aldrich) at concentrations of 1 ng/mL and 10 ng/mL, either for 12 h (for RNA isolation) or 24 h (for protein isolation, ELISA and exosome extraction). For exosome treatment, cultured HCECs were exposed to either M1 macrophage-derived exosome (M1 *exo*) or EGF-conditioned M1 macrophage-derived exosome (EGF-M1 *exo*) for 24 h.

### 4.8 Transmission electron microscope

For the observation of exosomes using transmission electron microscopy, the exosome pellet was initially fixed with 2.5 % glutaraldehyde and post-fixed with 2 % osmium tetroxide (OsO_4_) for 1 h. Subsequently, the exosome pellet was dehydrated using a graded series of acetone and embedded in the Spurr Low Viscosity Embedding Kit (#EM0300, Sigma-Aldrich). Ultrathin sections, approximately 65 nm thick, were prepared using an EM UC7 ultramicrotome (Leica). These sections were then placed on a copper grid, stained with uranyl acetate and lead citrate, and examined using a TEM (HT7800; Hitachi, Tokyo, Japan) at an accelerating voltage of 80 kV.

### 4.9 Proteomic analysis of exosomes by mass spectrometry

We established the protein profiles of exosomes isolated from macrophages with and without preconditioning with EGF using Liquid Chromatography (LC)/Mass Spectrometry (MS), as previously described [38]. In brief, we analyzed half of the digested sample through nano LC-MS/MS with a Waters NanoAcquity HPLC system connected to a ThermoFisher Q Exactive mass spectrometer. Peptides were loaded onto a trapping column and eluted over a 75 μm analytical column at a flow rate of 350 nL/min, employing a 2-hr reverse phase gradient. Both columns were packed with Luna C18 resin (Phenomenex). The mass spectrometer operated in data-dependent mode, with the Orbitrap running at 70,000 FWHM and 17,500 FWHM for MS and MS/MS, respectively. We selected the fifteen most abundant ions for MS/MS analysis.

Proteins were identified using a software (Proteome Discoverer™, Thermo Fisher Scientific) and compared against the human SwissProt protein databases. To assess the up-regulation or down-regulation of proteome among commonly identified proteins in M1 *exo*s and EGF-M1 *exo*s, and to quantify the extent of change, we utilized the relative abundance ratio (EGF-M1 *exo*/M1 *exo*). The proteome abundance data was obtained from the normalized values shown in Proteome Discoverer™ software. To explore the activated pathways between M1 *exo-*specific proteome and EGF-M1 *exo-*specific proteome, Reactome pathway analysis (https://reactome.org) was employed. We represented the top 25 pathways, ranked by the p-value. Subsequently, we screened all individual exosomal proteins in M1 *exo*s and EGF-M1 *exo*s, identifying immunologically relevant proteins. The determination of immunologically relevance relied on functional categories, including biological process from GO, Wiki pathway, and Reactome pathway information provided in Proteome Discoverer™ software. A protein was considered immunologically relevant if the annotated words in each category were directly or indirectly associated with immunology, inflammation, cell survival/death, and the related signals.

### 4.10 Purification of total RNA and Real-Time qRT-PCR

Total RNA was extracted from cells and mouse cornea tissue using the NucleoZOL (#740404.200, Macherey-Nagel, Germany) according to the manufacturer's instructions and quantitated using NanoDrop™ One (Thermo Fisher Scientific). Single-stranded complementary DNA (cDNA) was synthesized from 1 μg of total RNA using RevertAid First Strand cDNA Synthesis Kit (#K1622, Thermo Fisher Scientific). Real-time RT-PCR was conducted using a QuantStudio™ 3 Real-Time PCR System (Applied Biosystems™, CA, USA). Relative gene quantities were obtained using the comparative cycle threshold (Ct) method after normalization to 18S rRNA or glyceraldehyde-3-phosphate dehydrogenase (GAPDH) as a reference gene.

### 4.11 Western blot analysis

Total protein was isolated from cultured cells or exosome with RIPA buffer (#R0278, Sigma-Aldrich) supplemented with protease inhibitor cocktail (#11697498001, Roche, Basel, Switzerland) and phosphatase inhibitor (#4906845001, Roche). Lysates were incubated on ice for 15 min and centrifuged at 13,000 rpm at 4 °C for 15 min. Protein quantification using the Pierce™ BCA protein assay kit (Thermo Fisher Scientific). SDS-PAGE of protein was performed on 12.5 % gels. Proteins were transferred to nitrocellulose membranes (NC, Pall Corporation, New York, USA). Nonspecific antibody binding was blocked with 5 % skim milk in TBS-T (50 mM Tris-HCl pH 7.5, 150 mM NaCl, and 0.1% Tween-20) for 1 h at RT. Primary antibodies against EGF (#sc-275, Santa cruz,CA, USA), VEGF-A (#sc-7269, Sanata cruz), VCAM-1 (#MA5-31965, Invitrogen), IL-1β (#MBS423465, MyBioSource, Inc., CA, USA), IL-10 (#12163S, Cell Signaling Technology, MA, USA), CD63 (#10628D,Thermo Fisher Scientific), CD81 (#MA5-32333, Thermo Fisher Scientific), Hsp70 (#BML-SA660, Enzo Life Sciences, NY, USA) and β-actin (#sc-47778; Santa cruz) were diluted in antibody diluent (#003118, Thermo Fisher Scientific) and incubated overnight at 4 °C. Secondary antibodies diluted in TBS-T containing 5% skim milk (1:2500) were incubated for 1 h at RT. The value of each band was normalized to that of β-actin. The full-length gels before cropping in Fig. 2B and C and D are indicated in fig. S4 and fig. S5, respectively.

#### 4.11.1 ELISA

Concentrations of IL-6 in M0, M1 or EGF-treated M1 macrophage supernatants were measured using IL-6 ELISA development kits (#900-T16, PeproTech, NJ, USA) according to the manufacturer's instructions.

### 4.12 Flow cytometry

For plate-differentiated THP-1 cells, 1 × 10^6^ cells were added per tube. The cells were washed with cell staining buffer (1% BSA, 0.01% sodium azide in PBS) by centrifuging at 350×*g* for 5 min, and then we decanted the supernatant. The cells were blocked with 5 μL of human TruStain FcX (Fc receptor blocking solution; BioLegend) per 100 μL cell suspension for 10 min at RT, followed by a 20 min incubation in the dark and on ice, with the fluorochrome-conjugated anti-human antibodies (either anti-CD14 PE, anti-CD11b FITC, anti-CD11c APC or anti-CD14 PE, anti-CD86 APC, anti-CD163 FITC, anti-CD206 BV421). Following repeat of the wash step, and then resuspend cell pellet in 0.5 mL of cell staining buffer and add 5 μL of 7-AAD Viability staining solution (BioLegend) on ice in the dark. The cells were analyzed on flow cytometer (FACSCanto, BD BioSciences, Mountain View, CA, USA). The data was analyzed using FlowJo software (BD, Ashland, OR, USA).

### 4.13 Animal experiment

Twenty 6-week-old BALB/c male mice (Orient Bio Inc., Seongnam, Gyeonggi-do, Korea) were used. The corneal epithelium was removed using a No. 15 blade before applying exosome eye drops. The control group received 5 μL of PBS in one eye, while the exosome group received 30 μg/5 μL of M1 *exo*s diluted with PBS in the right eyes and 30 μg/5 μL of EGF (10 ng/mL)-M1 *exo*s diluted with PBS in the left eyes. Each eye received a total of 8 applications (3 times/day). After sacrificing the mice, corneal buttons were excised for analysis.

Mouse eye tissues were fixed in 4 % paraformaldehyde overnight, cryoprotected with 30% sucrose overnight, and embedded in optimal cutting temperature (OCT) compound (Tissue-Tek OCT Compound; Sakura Finetek, Torrance, CA) at −80 °C. Frozen OCT compound-embedded sections were cut at 8 μm thickness and placed on silane-coated microscope slides. For immunofluorescence, frozen sections were permeabilized with 0.3% Triton X-100 for 15 min at RT and then incubated with anti-F4/80 (Rat IgG diluted to 1:100, #123102, BioLegend) and anti-CD68 (Rabbit IgG1 diluted to 1:100, #PA5-89134 Thermo Fisher Scientific) at 4 °C. Sections were incubated with secondary antibodies (anti-rat IgG Alexa Fluor 568, anti-rabbit IgG Alexa Fluor 488, Thermo Fisher Scientific) for 1 h at RT. Slides were washed three times (5 min each) with PBS at each step. Cover slips were mounted on slides using Vectashield (Vector Laboratories, Burlingame, CA, USA) containing DAPI. The stained tissue was observed using an inverted microscope (DMi8, Laica).

### 4.14 Statistical analyses

Statistical analysis software (GraphPad Prism, ver. 10, GraphPad Software Inc., La Jolla, CA, USA) was used for statistical tests. To compare three or more groups, data were analyzed by the parametric ANOVA followed by Tuckey's post-hoc or non-parametric Kruskal-Wallis test followed by Dunn's *post-hoc*. To compare two groups, data were analyzed by the parametric Student's t-test or non-parametric Mann-Whitney *U* test. Data within graphs were presented as the mean ± standard error. Differences were considered statistically significant when *P* < 0.05.

## Data availability statement

The datasets generated and analyzed in the current study are available from the corresponding author on reasonable request.

## Funding disclosure

This work was supported by the by the 10.13039/501100003725National Research Foundation of Korea (NRF),South Korea grant funded by the Korean government (RS-2023-00209498, RS-2023-00219421).

## Ethics declarations

This study was reviewed and approved by the Institutional Review Boards at Chung-Ang University Hospital, with the approval number (2106-006-465).

## CRediT authorship contribution statement

**Soo Jin Lee:** Writing – original draft, Validation, Resources, Methodology, Conceptualization. **Seung Hyeun Lee:** Methodology, Investigation. **Ahra Koh:** Methodology, Investigation. **Kyoung Woo Kim:** Writing – review & editing, Writing – original draft, Supervision, Methodology, Investigation, Funding acquisition, Formal analysis, Conceptualization.

## Declaration of competing interest

The authors declare that they have no known competing financial interests or personal relationships that could have appeared to influence the work reported in this paper.
