# Supplementary material for: EGF-conditioned M1 macrophages Convey reduced inflammation into corneal endothelial cells through exosomes
Source: Heliyon. 2024 Feb 22;10(5):e26800. doi: 10.1016/j.heliyon.2024.e26800 (PMC10906407; doi:10.1016/j.heliyon.2024.e26800)
Supplement: Multimedia component 1 [file mmc1.pdf]

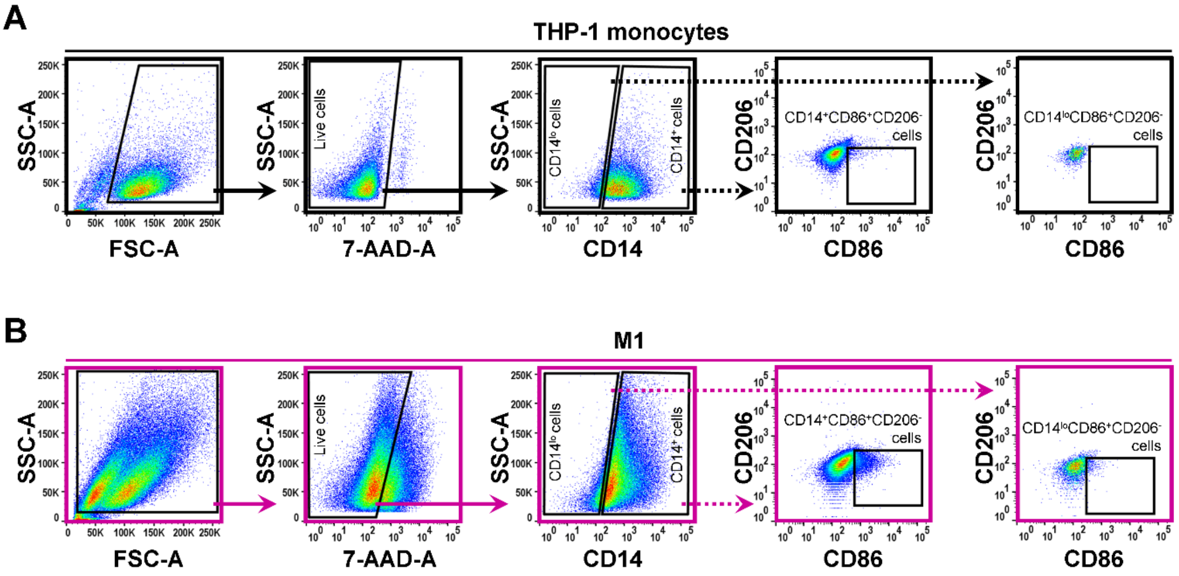

SUPPLEMENTARY FIGURE 1. Representative flow cytometric results for M1 macrophage polarization from THP-1 human monocyte cell line. Both CD14$^+$CD86$^+$CD206$^-$ and CD14^{lo}CD86$^+$CD206$^-$ cell populations which are relevant to M1 macrophages increased after the induction of M1 polarization (**B**) compared to before the induction (**A**).

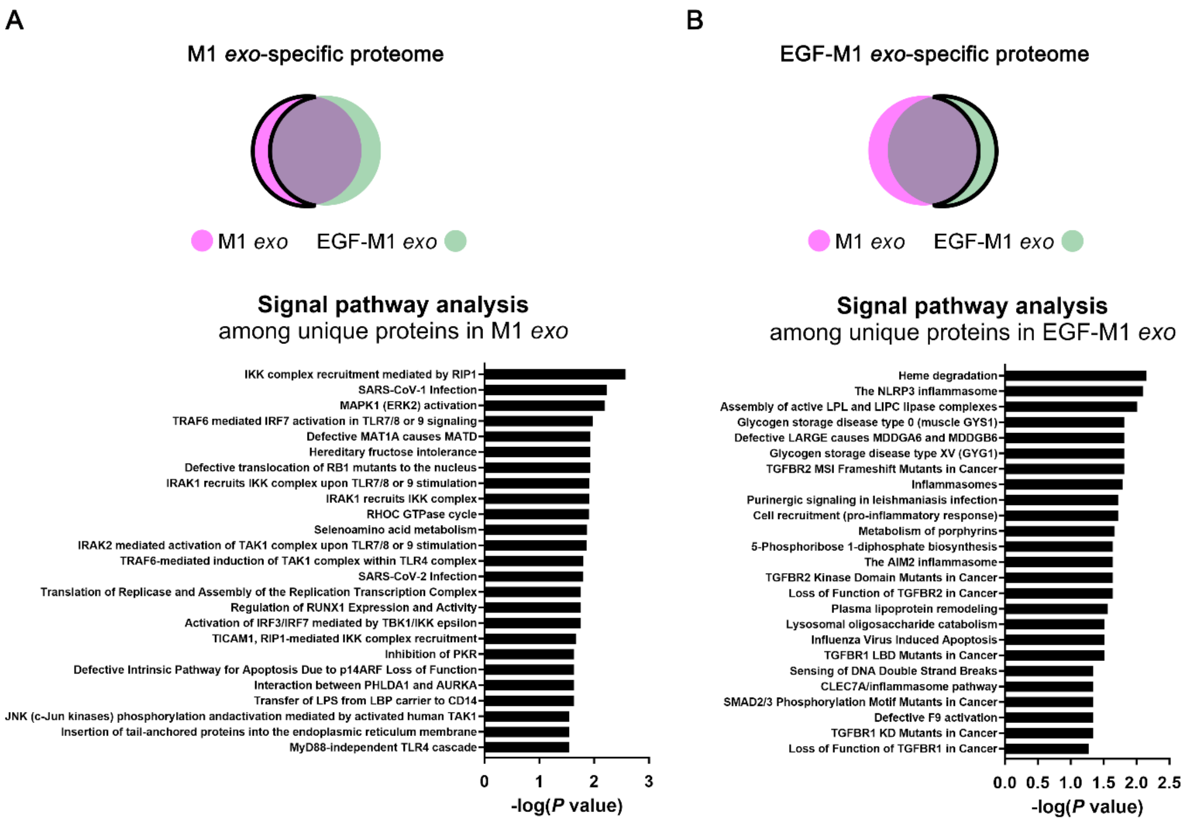

SUPPLEMENTARY FIGURE 2. EGF-induced activated signal pathways in the exosomal proteome derived from M1 macrophages. (**A-B**) The most significant 25 Reactome signal pathways in each M1 macrophage-derived exosome (M1 *exo*)-specific proteome (**A**) and EGF-conditioned M1 macrophage-derived exosome (EGF-M1 *exo*)-specific proteome (**B**).

A

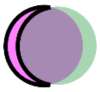

M1 *exo*-specific proteome

M1 *exo*   EGF-M1 *exo*

No	Protein	UniProt ID	Gene	Gene ontology biological process
1	**Interferon regulatory factor 3**	Q14653	***IRF3***	Immune system process, LPS-mediated signaling pathway, macrophage apoptotic process, positive regulation of inflammatory cytokines
2	**Tyrosine-protein phosphatase non-receptor type 11**	Q06124	***PTPN11***	Positive regulation of inflammatory cytokines, T cell co-stimulation
3	**Macrosialin**	P34810	***CD68***	Cellular response to LPS, Inflammatory response to antigenic stimulus
4	**Complement component 1 Q subcomponent-binding protein,mitochondrial**	Q07021	***C1QBP***	Complement activation (classic pathway), positive regulation of neutrophil chemotaxis
5	**Interferon regulatory factor 2-binding protein 2**	Q7Z5L9-1	***IRF2BP2***	Immature B cell differentiation
6	**Tumor necrosis factor**	P01375	***TNF***	Acute inflammatory response, positive regulation of inflammatory response and neutrophil activation
7	**TNF receptor-associated factor 6**	Q9Y4K3	***TRAF6***	Positive regulation of inflammatory response, TNF-mediated signaling pathway
8	**Vascular cell adhesion protein 1**	P19320-1	***VCAM1***	Acute inflammatory response, positive regulation of T cell proliferation, cellular response to TNF
9	**Sodium-dependent phosphate transporter 1**	Q8WUM9	***SLC20A1***	Positive regulation of I-kappaB kinase/NF-kappaB signaling

B

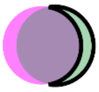

EGF-M1 *exo*-specific proteome

M1 *exo*   EGF-M1 *exo*

No	Protein	UniProt ID	Gene	Gene ontology biological process
1	**Collagen alpha-2(I) chain**	P08123	***COL1A2***	Transforming growth factor beta receptor signaling pathway
2	**Superoxide dismutase [Cu-Zn]**	P00441	***SOD1***	Negative regulation of inflammatory cytokines, removal of superoxide radicals
3	**Transforming growth factor beta-1**	P01137	***TGFB1***	Negative regulation of macrophage cytokine production
4	**Signal transducer and activator of transcription 2**	P52630-3	***STAT2***	Negative regulation of type I interferon-mediated signaling pathway

SUPPLEMENTARY FIGURE 3. Immune-related proteins specifically screened in M1 macrophage-derived exosomes and EGF-conditioned M1 macrophage-derived exosomes. (**A-B**) The screened proteins focused on proteins associated with immunological functions within each M1 macrophage-derived exosome (M1 *exo*)-specific proteome (**A**) and EGF-conditioned M1 macrophage-derived exosome (EGF-M1 *exo*)-specific proteome (**B**).

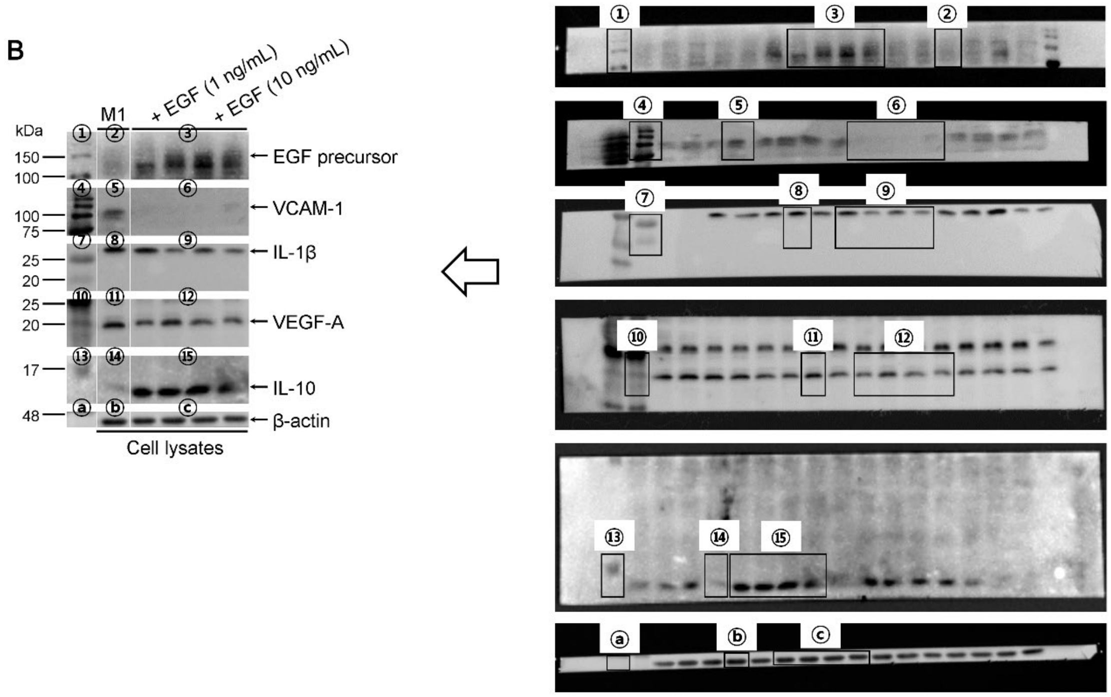

SUPPLEMENTARY FIGURE 4. Full-length gels in figure 2B before copping.

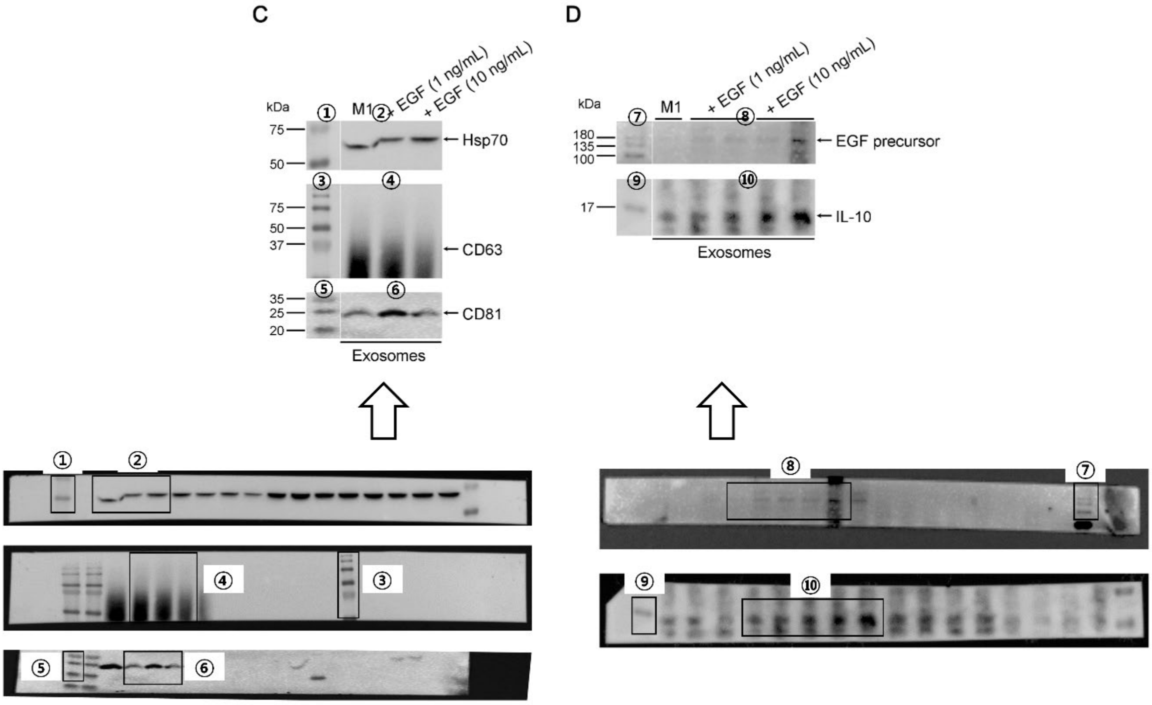

SUPPLEMENTARY FIGURE 5. Full-length gels in figure 4C and D before copping.
